# Modelling for policy: The five principles of the Neglected Tropical Diseases Modelling Consortium

**DOI:** 10.1371/journal.pntd.0008033

**Published:** 2020-04-09

**Authors:** Matthew R. Behrend, María-Gloria Basáñez, Jonathan I. D. Hamley, Travis C. Porco, Wilma A. Stolk, Martin Walker, Sake J. de Vlas, for the NTD Modelling Consortium

**Affiliations:** 1 Neglected Tropical Diseases, Bill & Melinda Gates Foundation, Seattle, Washington, United States of America; 2 Blue Well 8, Seattle, Washington, United States of America; 3 MRC Centre for Global Infectious Disease Analysis and London Centre for Neglected Tropical Disease Research, Department of Infectious Disease Epidemiology, Imperial College London, London, United Kingdom; 4 Francis I. Proctor Foundation for Research in Ophthalmology, Department of Epidemiology and Biostatistics, and Department of Ophthalmology, University of California, San Francisco, United States of America; 5 Department of Public Health, Erasmus MC, University Medical Center Rotterdam, Rotterdam, the Netherlands; 6 London Centre for Neglected Tropical Disease Research, Department of Pathobiology and Population Sciences, Royal Veterinary College, Hatfield, Hertfordshire, United Kingdom; 7 London Centre for Neglected Tropical Disease Research and Department of Infectious Disease Epidemiology, Imperial College London, London, United Kingdom; Centers for Disease Control and Prevention, UNITED STATES

## Introduction

The neglected tropical diseases (NTDs) thrive mainly among the poorest populations of the world. The World Health Organization (WHO) has set ambitious targets for eliminating much of the burden (and the transmission when possible) of these diseases by 2020 [1], with new targets for 2030 being currently set [2]. Substantial international investment has been made with the London Declaration (2012) on NTDs to prevent the morbidity and premature mortality associated with these diseases through global programmes for their control and elimination.

The NTD Modelling Consortium [3] is an international effort to improve the health of the poorest populations in the world through the development and application of mathematical (including statistical and geographical) models for NTD transmission and control.

Although policy and intervention planning for disease control efforts have been supported by mathematical models [4–6], our general experience is that modelling-based evidence still remains less readily accepted by decision-making bodies than expert opinion or evidence from empirical research studies. Toward increasing modelling impact, we (1) conducted a review of the literature on (health-related) modelling principles and standards, (2) developed recommendations for areas of communication in policy-driven modelling to guide NTD programmes, and (3) presented this to the wider NTD Modelling Consortium.

Principles were formed as a guide for areas to communicate the quality and relevance of modelling to stakeholders. It is not guidance for communicating models to other modellers or how to conduct modelling. In adhering to a practise of these principles, our hope is that modelling will be of greater use to policy and decision makers in the field of NTD control, and possibly beyond that.

### Examples of successes in modelling for policy in the field of NTDs

We first wish to recognise some of the successful examples of NTD programme relationships with modellers. The motivation for employing principled communication, as we propose, is to deliver a similarly positive impact consistently over time and for different NTDs. Onchocerciasis (a filarial disease caused by infection with *Onchocerca volvulus* and transmitted by blackfly, *Simulium*, vectors) probably provides the best example of impactful modelling, with its long history of using evidence—mostly from the ONCHOSIM and EPIONCHO transmission models [7]—to support decision-making within ongoing multicountry control initiatives (Table 1).

**Table 1:** Onchocerciasis modelling and policy impact.

Specific public health challenge	How modelling addressed the challenge
What is the minimal duration of the OCP necessary to mitigate the risk of recrudescence after cessation of interventions?	ONCHOSIM guided duration of vector control operations in the OCP and investigated the combined impact of vector and ivermectin treatment to reduce programme duration (1997) [5].
What is the feasibility of reaching elimination of onchocerciasis transmission based on ivermectin distribution as the sole intervention (i.e., in the absence of vector control)?	ONCHOSIM informed the Conceptual and Operational Framework of Onchocerciasis Elimination with Ivermectin Treatment launched by the APOC (2010) [8], and EPIONCHO and ONCHOSIM were fitted to data from proof-of-principle elimination studies in foci of Mali and Senegal (2017) [9].
Areas where onchocerciasis–loiasis are coendemic present challenges for ivermectin treatment because of the risk of SAEs in individuals with high *Loa loa* burden.	Environmental risk modelling helped to guide distribution of ivermectin by mapping risk for *L*. *loa* coendemicity in Cameroon (2007) [10].
	Geostatistical mapping, based on RAPLOA data in 11 countries, informed where extra precautionary methods or alternative strategies are needed to minimise SAE risk (2011) [11].
Annual ivermectin distribution may not be sufficient to achieve elimination in foci with high baseline (precontrol) endemicity.	EPIONCHO and ONCHOSIM supported the shift to 6-monthly ivermectin treatment in highly endemic foci in Africa (2014) [12, 13].
At the closure of the APOC in 2015, there was a need to delineate current and alternative/complementary intervention tools to reach elimination at the continental level.	EPIONCHO and ONCHOSIM supported deliberations and final APOC’s report on Strategic Options and Alternative Treatment Strategies for Accelerating Onchocerciasis Elimination in Africa (2015) [6].
Drug discovery and clinical trial design and analysis are essential toward optimising alternative treatment strategies based on the use of macrofilaricides (drugs that kill adult *O*. *volvulus*).	Modelling facilitated analysis of clinical trials and informed drug discovery and development by the A∙WOL Consortium (2015–2017) [14, 15].

Abbreviations: APOC, African Programme for Onchocerciasis Control; A∙WOL, Anti-Wolbachia; OCP, Onchocerciasis Control Programme in West Africa; RAPLOA, Rapid Assessment of Prevalence of Loiasis; SAE, severe adverse event

From the start of the NTD Modelling Consortium in 2015, there have been several other examples of impactful modelling, which could be divided over three major scales of operations: (1) developing WHO guidelines (e.g., for triple-drug therapy, with ivermectin, diethylcarbamazine, and albendazole, against lymphatic filariasis [16, 17]); (2) informing funding decisions for new intervention tools (e.g., the development of a schistosomiasis vaccine [18]); and (3) guiding within-country targeting of control (e.g., local vector control for human African trypanosomiasis in the Democratic Republic of the Congo [19, 20] and Chad [21]).

## Methods

### Literature review

Our review aimed to inform the present synthesis of principles for the consortium. We evaluated published guidelines for good modelling practises in health-related modelling through a review and qualitative synthesis, following a systematised approach. We searched Equator Network Library for Health Research Reporting and PubMed with terms targeting guidance and good practises for mathematical modelling in the area of human health. The PubMed search applied the systematic[sb] filter with title-and-abstract terms (guideline* OR guidance OR reporting OR checklist OR ((best OR good) AND practice*))) AND model* NOT animal, plus any one of a combination of common modelling terms occurring in the full text. The full search strategy is described in S1 Appendix (Literature review search strategy and Search strategy and selection criteria). Studies in the form of reviews and guidelines were eligible for consideration, and those discussing modelling in the abstract or title were included. Results were then expanded by including references included in recent systematic and rapid reviews [22, 23]. Succinct statements were extracted for analysis, excluding elaborative text. Text was copied and pasted from PDF files to standardised study extraction spreadsheets.

**Fig 1:**
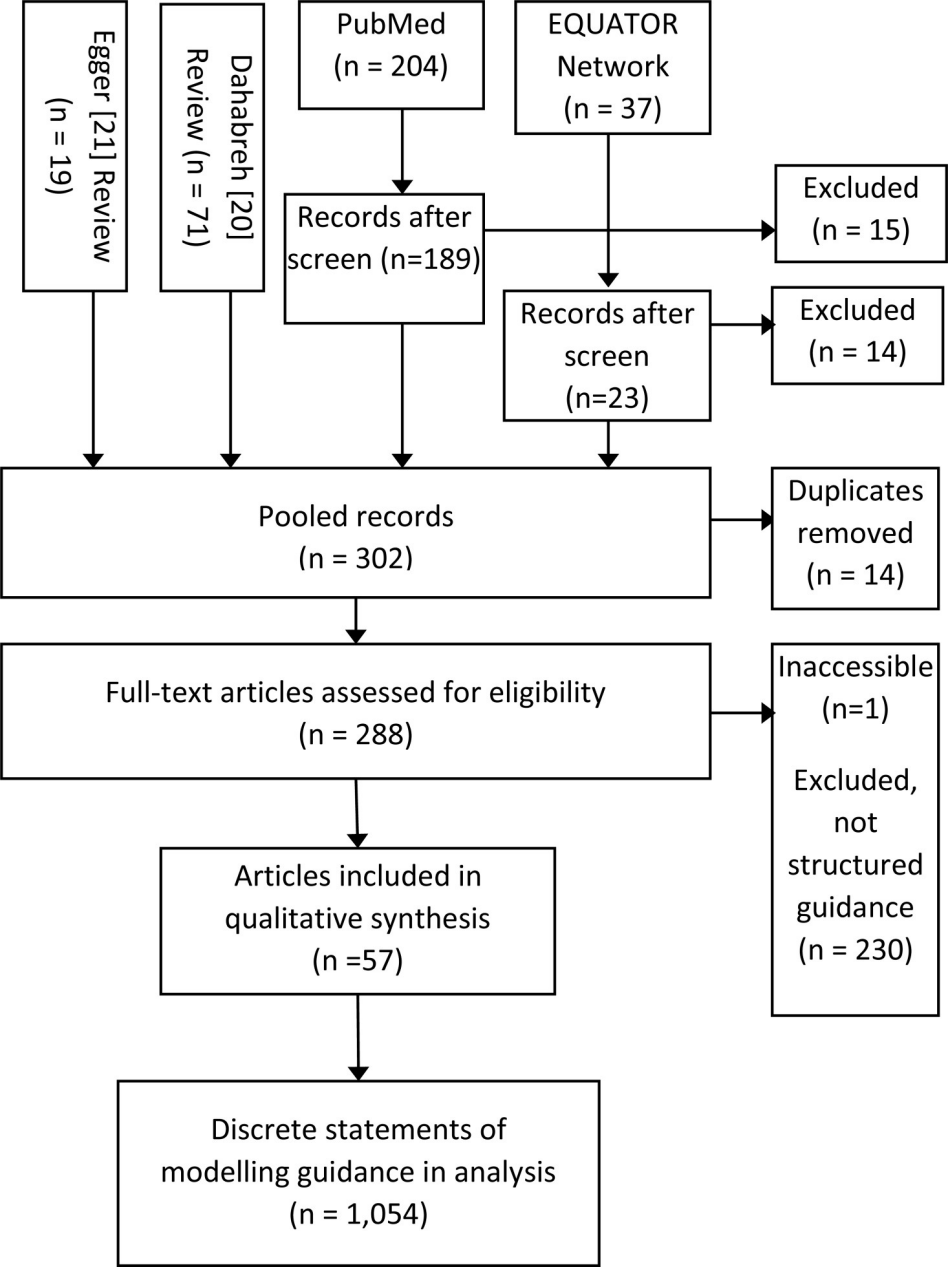
Study selection.

**Fig 2:**
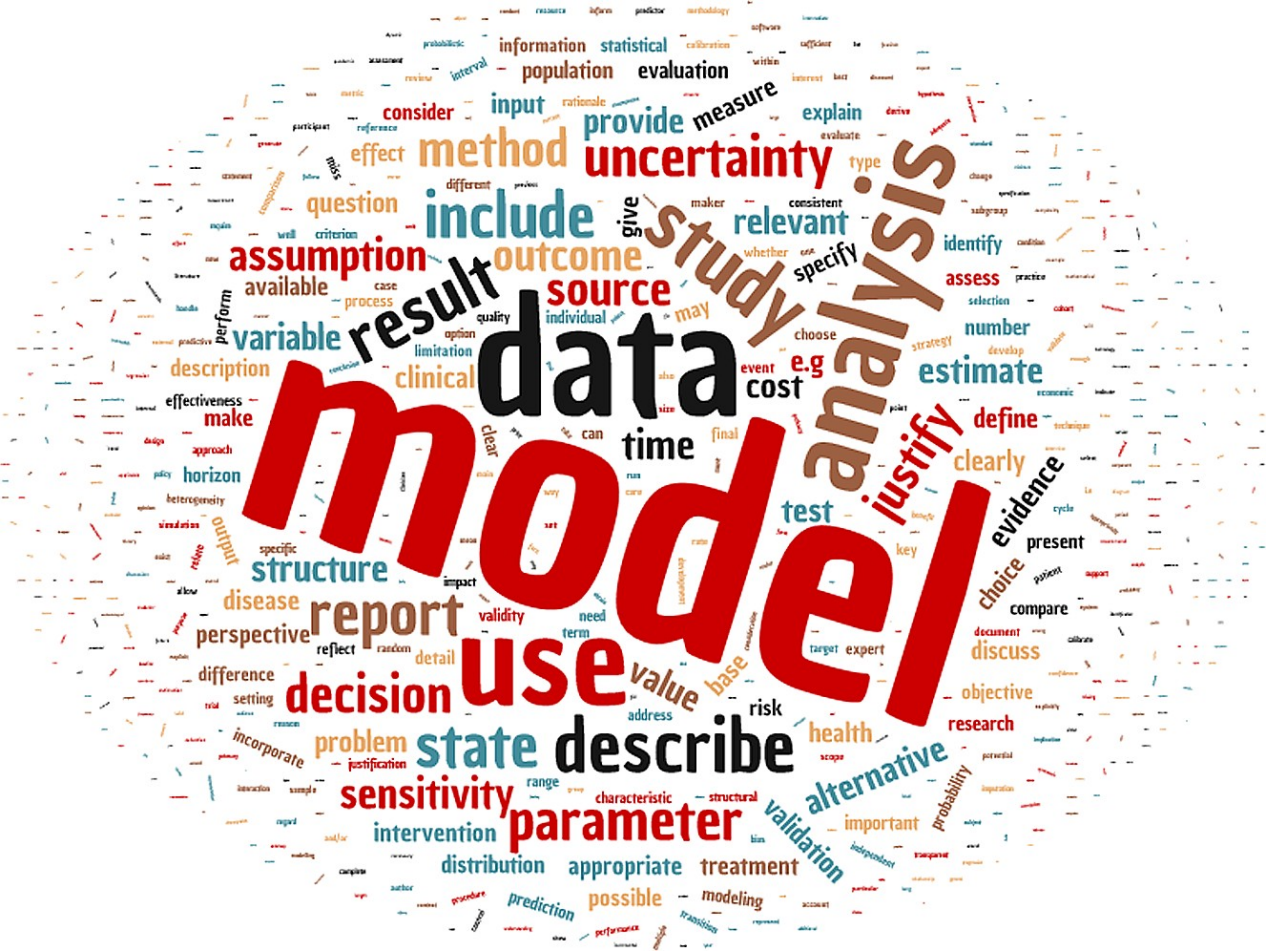
Word cloud of the 1,054 modelling guidance statements. Relative word frequencies are represented by size of the font.

We identified 288 studies relevant to modelling practises, of which 57 were included [24–80] (Fig 1). See S1 Appendix (Table 1) for characteristics of included studies. Studies in the form of reviews and guidelines were eligible for consideration, and those discussing modelling conduct or reporting in the abstract or title were included. Studies were excluded if guidance to modellers was not presented in a list or table to facilitate inspection. However, exclusions were most frequently due to absence of guidance to modellers rather than because guidance was not provided in a structured format. Altogether, included studies contained 1,054 succinct statements of modelling guidance that were included in the qualitative synthesis. A summary of the data set contents is given visually (Fig 2) and as a table of word occurrence counts in S1 Appendix (Table 2).

### Scoring the guidance statements

Authors coded the data set individually (MRB, TCP, WAS, SJdV) and jointly (M-GB, JIDH, MW), producing five independently coded sets of data (S1 Table). Modelling guideline statements were coded with the following ordinal scale of importance scores: 1, not applicable; 2, not necessary; 3, important; 4, extremely important; and 5, obvious (i.e., merely restates principles regarded as universally agreed upon; see S1 Table).

In coding the data set, we saw that many of the 1,054 statements rephrased the same concepts (e.g., ‘do an uncertainty analysis’). Statements similar in meaning could be given different scores simply because they were phrased differently (S1 Appendix, Interrater reliability).

We rank-sorted statements by score to select the top few statements we collectively considered extremely important. A group of 46 statements consistently received a score of 4 (extremely important) from at least four of the authors. The most important 46 ranked statements were selected, evaluated for content, and gradually categorised into five major themes (S2 Table) through discussion. In each theme, we formulated a single principle that distilled the statements grouped under that theme. Original text for each statement was preserved up to this final stage of our synthesis. Preliminary formulations of the principles were discussed with a subset of the larger NTD Modelling Consortium group at a meeting in New Orleans (28 October 2018) and further refined for presentation at the consortium Technical Meeting in Oxford (20 March 2019).

Five of the 46 guidance statements at the top of our ranked list did not fit well into the categories we settled upon for principles. From those that did not become part of a principle, we formed two philosophies that reflect some of our ideals. These will be presented in the Discussion.

## Results

### Consortium principles

Five principles (Box 1) are the results produced by our distillation and synthesis of guidance on good modelling practises we found in the literature. Adoption of these principles as consortium principles is a result of about 2 years of engagement with the consortium membership. See section Principles in practise for how adherence might be demonstrated.

**Box 1: The five principles of the NTD modelling consortium**

1. Don't do it alone. Engage *stakeholders throughout*, from the formulation of questions to the discussions on the implications of the findings.
2. *Reproducibility* is key! Prepare and make available (preferably as *open-source* material) a complete technical *documentation* of all model code, mathematical formulas, and assumptions and their justification, allowing others to reproduce the model.
3. Data play a critical role in any scientific modelling exercise. All *data* used for model quantification, calibration, goodness of fit, or validation should be described in *sufficient detail* to allow the reader to assess the type and quality of these analyses. When referencing data, apply Principle 2.
4. Communicating uncertainty is a hallmark of good modelling practise. Perform a *sensitivity analysis* of all key parameters, and for each paper reporting model predictions, include an *uncertainty assessment* of those model outputs within the paper.
5. Model outcomes should be articulated in the form of testable hypotheses. This allows comparison with *other models* and *future events* as part of the ongoing cycle of model improvement.

### Principle 1: Stakeholder engagement

Policy makers and other stakeholders should be involved early and throughout the process of developing a model. Stakeholder engagement helps to ensure that the right balance is achieved between what decision makers and practitioners want and what modellers should and can provide to ensure that realistic policy options are being analysed and that proposed strategies for disease control are culturally or socially acceptable. The process of distilling what modelling needs to provide takes time to accomplish through dialogue. Stakeholders are essential to ensure the best available knowledge and evidence are used in model design, calibration, and validation. Finally, stakeholders are essential to interpret, translate, and integrate the implications of the findings.

Inclusion of stakeholders as authors in publications is important, including modeller stakeholders. Lack of trust in modelling studies partly reflects limited representation of modelling expertise from NTD-affected countries. The modelling community needs to support more local development of capacity for modelling and make sure that local technical capacity is genuinely engaged in discussions. Science on NTDs is increasingly changing in a positive way in this respect, but modelling has a longer way to go on this.

Building confidence in a model’s usefulness is a gradual process [81]. For this reason, we suggest that modelling studies choose to involve stakeholders early, ideally from the planning phase [82]. We believe that models that are considered to be jointly owned by modellers and stakeholders have a higher chance of becoming impactful for policy. Of course, at times, some stakeholders may not desire involvement of modelling teams, perhaps due to differences in perspective or even conflicts of interest, but stakeholder involvement in model development should remain a primary goal.

### Principle 2: Complete model documentation

An analysis should be described in sufficient detail for others to be able to implement it and reproduce the results [83]. Striving for this degree of clarity and transparency is good for reproducibility [84, 85] and also motivates changes in conduct to raise quality [86, 87]. A protocol often used to document agent-based models has shown success in raising their rigour [88]. Open-source software is only the first step of documentation. Deterministic and stochastic models need to present the equations, diagrams, and event tables that describe their behaviour. Agent-based models require more attention to completeness to be clear about what events can happen to heterogeneous individuals and according to which probability distributions.

Information (data and code) generated in modelling should be maintained according to common good software practises [89, 90] to ensure longevity [91], ideally on data-sharing platforms [92]. The funding and resources for doing this maintenance could be considered when planning the projects. In computational practises [90], ‘…decision makers who use results from codes should begin requiring extensive, well documented verification and validation activities from code developers’. Perfection is not the goal here, but thoughtful practises. Academic groups can transfer practical experience [89], so good computational practises also belong in our discourse. We invite stakeholders to ask each other, and to ask modellers, which quality controls are protecting the integrity of the modelling work.

### Principle 3: Complete description of data used

It should be understandable how empirical data and evidence were used (or not used) for model calibration, goodness-of-fit assessment, and partial validation (partial because models are typically used to predict policy outcomes for which sufficient empirical data are not always available). Employed data sets should be clearly described to allow readers to assess their quality and informativeness for specific model assumptions. The relevant context of data collection should additionally be communicated along with model results. Descriptions of employed data sets are central to building confidence in various assumptions in the model design. Model assumptions may be justified by support from data, and when key assumptions do gain acceptance conditioned on data, they must be reconsidered with multiple data sets. If the assumptions are valid, they should continue to be supported by new data sets over time, which may also lead to further dialogue on data requirements, before a model can be used to predict new scenarios. New information may dictate alterations to a model.

Calibration and validation are crucial for determining how well the model has been specified and parameterised, guiding identification of key processes that should be included in order to capture phenomena identified through model fitting to retrospective data and/or through forecasting. Principle 3 helps us to assess parametric assumptions and model analyses, as they may be limited by input data quality, and to identify data gaps and/or essential processes that may lead to reformulation of structural assumptions.

### Principle 4: Communicating uncertainty

Robust decisions are likely to be successful in the face of future uncertain events. Arguably one of the most useful contributions of a model is to estimate how much uncertainty the future may hold so that decisions may reasonably balance cost with risk. Therefore, stakeholders might expect to receive a clear presentation of uncertainty relative to the decision problem. Broad categories of uncertainty sources might be classed as fitted parameters, data inputs, model structure, and stochasticity.

‘As with experimental results, the key to successfully reporting a mathematical model is to provide an honest appraisal and representation of uncertainty in the model’s prediction, parameters, and (where appropriate) in the structure of the model itself’ [93]. A sensitivity analysis will demonstrate which parameters (or combinations of parameters) are most important for the outcome of interest, thereby indicating for which parameters proper quantification based on high quality data is most essential. By using realistic assessments of uncertainty in parameter values and structural assumptions (i.e., parametric and structural uncertainty), it should then be demonstrated in a so-called robustness or uncertainty analysis how the model outcome is subject to overall uncertainty.

A consortium is a good forum (as exists for, among others, HIV, malaria, and NTDs) to understand structural uncertainties between multiple modelling groups, including reducing the overall level of uncertainty by ensembles [94] or other means of combining models. Openness in assumptions can further help assessing the impact of poorly understood sources of uncertainty on outcomes; for example, parameters called ‘fixed’ (i.e., an assumed value) may need assessment, as well as assumptions about the fundamental processes underlying data patterns. Modellers should excel in transparency of how uncertainty was estimated, and stakeholders should not accept a projection without uncertainty bounds.

### Principle 5: Testable model outcomes

Specific challenges to the use of forecasting arise in a policy context. Nevertheless, prediction and falsification are of central importance in science [95, 96]. The life span of a model is typically long, and over time, the same model may be applied to different policy questions. Model validation thus becomes an ongoing process. Models are often used to predict future trends in infection and draw conclusions on specific policy questions in the absence of data. However, data may become available at a later time and should then be used to further validate the model, leading to a better model and more confidence in its predictions. Moreover, when possible, forecasts may be made for a range of scenarios outside those for which data will be collected, as data collection programmes may be expanded. Modelling studies aiming at defining a threshold or the most cost-effective strategy should also present expected future trends for situations in which this threshold or strategy would actually be applied so that these trends can potentially be compared with future data and proposed thresholds or strategies can be reevaluated if necessary, or the context in which they apply can be better defined and understood.

Model comparison, one of the main activities of the NTD Modelling Consortium [97], requires multiple independent modelling groups working on each disease to explain collaboratively any differences between their model results on that disease. Agreement on a weighting method allowing for an ensemble [98, 99], or otherwise placing results in a coherent framework, supports clear interpretation of all results. Model comparisons are generally best done in a masked manner, with data partitioned into a training set and a test set. A sufficient sample size, together with probabilistic forecasting with proper scoring [100], can be used in forecast comparisons, permitting objective and falsifiable comparisons. In looking to apply a model to new or future problems, models cannot be truly ‘validated’ for a future scenario outside of their training conditions, but an open and transparent collection of models, which have survived efforts at prospective testing, can provide more confidence in their prospective policy analyses. Forecasting is garnering increasing interest outside NTDs, as shown by the Centers for Disease Control and Prevention (CDC) sponsorship of an annual forecasting contest for the United States influenza-like illness data [101, 102]. Guidelines for structured model comparisons were recently proposed to improve the quality of information available for policy decisions [103]. Stakeholders can help build trust for objective comparison exercises by promoting right incentives for inclusive comparisons.

Finally, we conjecture two additional benefits of objective testing that might be communicated: (1) helping to avoid the danger of excessive agreement and ‘groupthink’—a failure to challenge conventional wisdom with a truly searching inquisition, and (2) helping avoid to bias.

### Principles in practise: Policy-relevant items for reporting models in epidemiology of neglected tropical diseases summary table

How can these five principles be upheld in practise? The principles are alive and well when we regularly engage each other on demonstrations of the principles, express them in our publications, and demonstrate them in relationships with our stakeholders. Principles identify broad themes that modellers should consider when reporting and communicating their research findings. The reason we do this is to properly support the success of our stakeholders in making use of modelling evidence.

We offer a summary table as a simple tool to write how each principle was fulfilled, or perhaps what challenges were found. We call it the Policy-Relevant Items for Reporting Models in Epidemiology of Neglected Tropical Diseases (PRIME-NTD) Summary Table (Table 2 and S1 Appendix). It is a means to promote engagement with the principles and to improve accessibility, communication, and reporting of modelling results. We promise to show our stakeholders how we demonstrated the principles for them in a summary table to be included with presentations and publications on policy questions. We recommend more broadly that modellers follow a similar approach when making results available for policy matters. Stakeholders are then invited to verify that the principles are in fact used in the modelling studies.

**Table 2:** PRIME-NTD summary table.

Principle	What has been done to satisfy the principle?	Where in the manuscript is this described?
**1. Stakeholder engagement**		
**2. Complete model documentation**		
**3. Complete description of data used**		
**4. Communicating uncertainty**		
**5. Testable model outcomes**		

## Discussion

Although many guidelines on modelling are already available [22, 23, 64], they are often not implemented in practise. As part of an overall commitment to evidence-based decision-making, we have reaffirmed existing recommendations regarding reproducibility, fidelity to data, and accurate communication of uncertainty. We also found it important to extend existing recommendations to emphasise the importance of stakeholder involvement (Principle 1) and predictive testing [43, 104] (Principle 5). Stakeholder involvement can bring epidemiological expertise, analytic relevance, and ultimately richer data. Striving for predictive testing by providing forward projections can provide a sharper model test than one that fits to existing data alone, and it reflects a commitment to hypothesis testing and the scientific method. What makes our contribution notable is that we are adopting the guidance ourselves and making a commitment to our stakeholders that we are accountable to demonstrate our principles throughout engagement.

**Fig 3:**
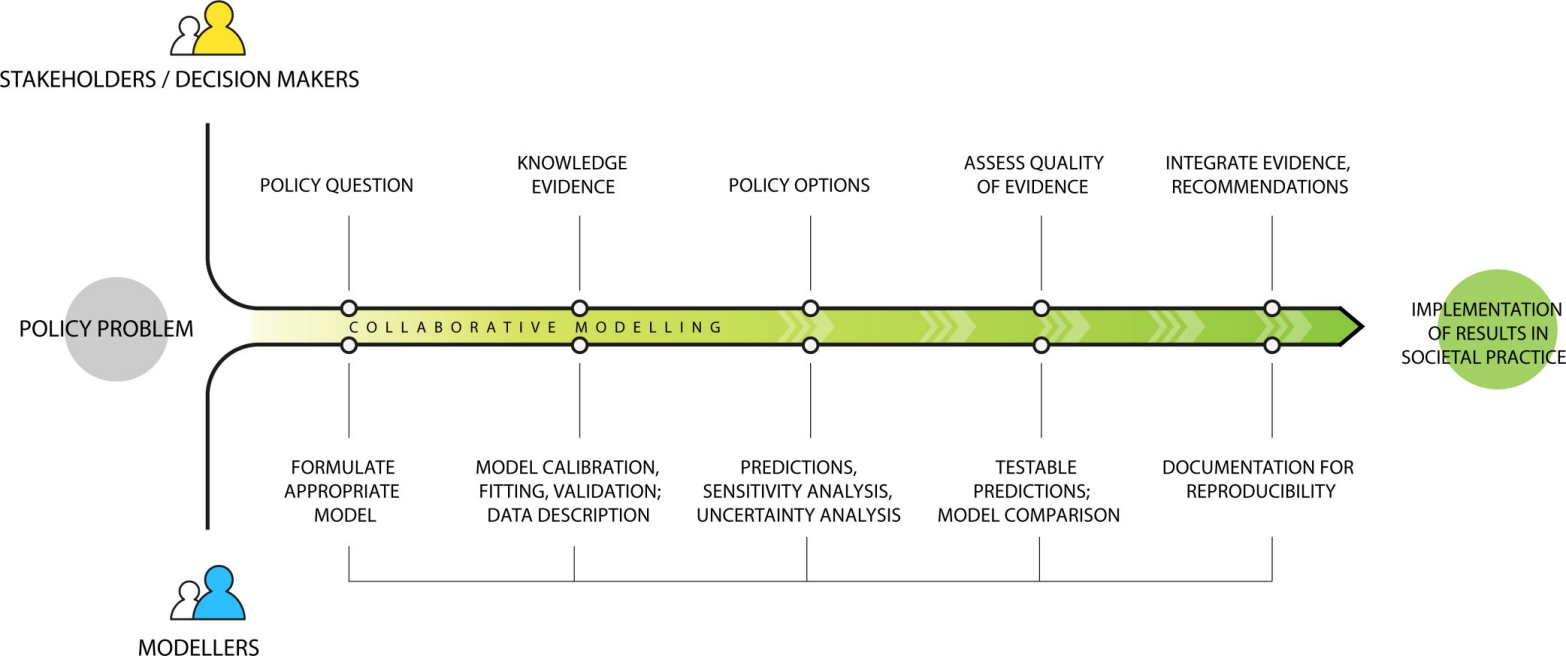
Illustration of a collaborative process between modellers and stakeholders/decision makers. Each group brings something to the table at different time points. The best modelling result with eventual impact is usually only obtained in collaboration. Although the process is depicted as linear, in practise each node may connect back to stakeholders and modellers for continuous dialogue and discussion; policy implementation can also be reevaluated in light of evidence.

Dialogue with stakeholders can help to improve the quality and responsiveness of quantitative efforts to assess and inform health policy [105]. From formulating questions toward results and potentially to implementation, the timing and nature of feedback should follow some arranged plan for engagement that is not left to chance or whim. Fig 3 shows an example of a collaborative process.

From the review, we also arrived at two philosophies that reflect some of our ideals. The first is that modelling is an ongoing process, i.e., models should never be regarded as complete or immutable. They should be repeatedly updated, and sometimes abandoned and replaced, as new evidence or analyses become available to inform their structure or input. The second philosophy is that the NTD Modelling Consortium strives for a mechanistic formulation of models whenever possible. This means incorporating into the models processes underlying transmission and realistic operational contexts to measure things the same way a control or research programme measures them. Moreover, the needs of public health and policy, we believe, favour a mechanistic approach that permits testing counterfactual scenarios and helps in communication with lay, nonmathematical stakeholders.

Depending on the perspective, it may be a limitation of our study that key stakeholders outside our consortium are not included as coauthors of our piece. By design, this work represents our consortium’s understanding of what stakeholders have been asking us to do over the course of ongoing engagements. Also a limitation of our review and qualitative synthesis is that modelling fields outside of health were not searched, though they often relate well to the modelling of diseases. The review was designed to thoroughly cover concepts appearing in modelling guidance. It is not comprehensive of guidance issued. We abstracted some potential indicators of future practise, such as having a statement of adherence (S1 Appendix—Table 2), but we did not attempt to assess the use of guidance following their publication.

Guidelines for evidence synthesis allow unbiased integration of evidence in high-stakes controversial settings [106]. Our study enhances communication required for properly evaluating models, which complements recent initiatives by WHO on decision-making frameworks inclusive of mathematical models [23, 107], qualitative systematic reviews [108], and operational research [109]. These frameworks extend the Grading of Recommendations Assessment, Development and Evaluation (GRADE) [110]. Expert groups such as WHO Initiative for Vaccine Research sometimes evaluate models to support evidence synthesis, but there is yet no standard way to integrate modelling into WHO guidelines development as there is for clinical evidence [111]. One motivation for extending existing guidelines is that understanding risk of bias in models [23, 31, 112] cannot be done well using the same approaches to bias risk assessment for empirical studies.

The need for guidelines has been well established [113], which has led to accepted and practised standards for health research [114]. In this review, we found that only four [30, 38, 41, 45] of 57 guideline proposals had recognisable statements of commitment to their recommendations such that the authors or others were actively encouraged to follow them. There may be more adherents, but initial commitment is a striking indicator consistent with utilisation of modelling guidance [37]. Additional successfully established modelling guidelines exist, for example, on describing agent-based models [115] in theoretical ecology. In this example, the authors later conducted a review of studies applying their guidelines [88], updating them based on ongoing discussions with those who had adopted them to improve clarity and avoid redundancy. A subtle outcome of our own work was that the process of synthesis was important for the authors. Ongoing discussion throughout the synthesis process was shaped by our intent to adopt the principles, which allowed a better understanding of how these might be practised and of any potential barriers that might be encountered in doing so.

In conclusion, we believe that by distilling the five principles of the NTD Modelling Consortium for policy-relevant work, and communicating our adherence to them, we will improve as modellers over time and enjoy more effective partnerships in the meantime. We ask our stakeholders to hold us to our promise. We also believe that the impact of applied modelling in other fields may benefit from doing the same.

## Supporting information

S1 Appendix PRIME-NTD Summary Table and methods detail. PRIME-NTD, Policy-Relevant Items for Reporting Models in Epidemiology of Neglected Tropical Diseases.
(DOCX)

S1 Table Excel file for the list of all 1,054 modelling guidance statements. (XLSX)

S2 Table Excel file for top 46 modelling guidance statements, grouped in themes. (XLSX)
