# Supplementary material for: Modelling for policy: The five principles of the Neglected Tropical Diseases Modelling Consortium
Source: PLoS Negl Trop Dis. 2020 Apr 9;14(4):e0008033. doi: 10.1371/journal.pntd.0008033 (PMC7144973; doi:10.1371/journal.pntd.0008033)
Supplement: S1 Appendix — PRIME-NTD, Policy-Relevant Items for Reporting Models in Epidemiology of Neglected Tropical Diseases. (DOCX) [file pntd.0008033.s001.docx]

Supplementary Appendix

**Modelling for policy: the five principles of the Neglected Tropical Diseases Modelling Consortium**

Matthew R. Behrend^1^ · María-Gloria Basáñez · Jonathan I. D. Hamley · Travis C. Porco ·
Wilma A. Stolk · Martin Walker · Sake J. de Vlas · for the NTD Modelling Consortium

^1^ behrend04@gmail.com

Table of Contents

[PRIME-NTD Summary Table 2](#_Toc15430762)

[1. Literature review search strategy 3](#_Toc15430763)

[2. Search strategy and selection criteria 4](#_Toc15430764)

[3. Data analysis methods 4](#_Toc15430765)

[4. Results 4](#_Toc15430766)

[Table 1 Characteristics of included studies 5](#_Toc15430767)

[5. Interrater reliability 7](#_Toc15430768)

[6. Word occurrence counts in guidance statements 7](#_Toc15430769)

[Table S Word occurrence counts in guidance dataset 7](#_Toc15430770)

[7. References to the supplementary appendix 9](#_Toc15430771)

## **PRIME-NTD Summary Table**

| Reference to the work summarized: |  |
| --- | --- |

| Principle | What has been done to  satisfy the principle? | Where in the manuscript is this described? |
| --- | --- | --- |
| 1. **Stakeholder engagement** |  |  |
| 1. **Complete model documentation** |  |  |
| 1. **Complete description of data used** |  |  |
| 1. **Communicating uncertainty** |  |  |
| 1. **Testable model outcomes** |  |  |

Full formulation of the principles:

1. Don't do it alone. Engage stakeholders throughout, from the formulation of questions to the discussions on the implications of the findings.
2. Reproducibility is key! Prepare and make available (preferably open-source) a complete technical documentation of all model code, mathematical formulas, assumptions and their justification, allowing others to reproduce the model.
3. Model calibration, goodness-of-fit and validation are fundamental processes of scientific modelling. All data used should be described in sufficient detail to allow the reader to assess the type and quality of these analyses. When using data by reference, use Principle 2.
4. Communicating uncertainty is a hallmark of good modelling practice. Perform a sensitivity analysis of all key parameters, and for each paper reporting model predictions include an uncertainty assessment of those model outputs within the paper.
5. Model outcomes should be articulated in the form of testable hypotheses. This allows comparison with other models and future events as part of the ongoing cycle of model improvement.

Incorporating prior work by reference in the paper is sufficient. Write what relevant information is contained in the prior work; then note its reference number in the summary table. Please verify that the whole chain of steps in referenced work is actually complete and up-to-date.

## **Literature review search strategy**

Search PubMed with the combined set of terms:

systematic[sb]

AND (guideline*[tiab] OR guidance[tiab] OR reporting[tiab] OR checklist[tiab] OR ((best[tiab] or good[tiab]) AND practice*[tiab]))

AND model*[tiab]

NOT animal[tiab]

AND (mathematic*[tw] OR predict*[tw] OR disease[tw] OR forecast*[tw] OR validat*[tw])

AND (uncertaint*[tw] OR confidence[tw] OR credible[tw] OR sensitivit*[tw])

AND (parameter*[tw] OR assumption*[tw] OR calibrat*[tw] or applicab*[tw] OR structur*[tw])

Search the EQUATOR listing (<http://www.equator-network.org/reporting-guidelines/>), using each of one of four terms:

model
models
modeling
modelling

PubMed and EQUATOR databases were searched 11 Jan 2018.

Study selection and data extraction:

Screen abstracts of records returned from each database to include articles broadly on mathematical modelling

Additionally, include all studies included in reviews by Dahabreh et al (2017) [[1](#_ENREF_1)] and Egger et al (2017) [[2](#_ENREF_2)].

Exclude duplicate records

Exclude studies by full text without a list or table of guidance on the conduct or reporting of modelling

Extract text from the list or table of guidance in the PDF file of the article

For each item/cell, observing cues given by font and formatting,

Highlight and copy text of a succinct statement, excluding any elaborative text

Paste the succinct statement to a new row in a study extraction spreadsheet for that article

## **Search strategy and selection criteria**

We searched Equator Network Library for Health Research Reporting and PubMed with terms targeting guidance and good practices for mathematical modelling in the area of human health. Key search terms were (systematic[sb] AND (guideline*[tiab] OR guidance[tiab] OR reporting[tiab] OR checklist[tiab] OR ((best[tiab] or good[tiab]) AND practice*[tiab]))) plus one of a combination of common modelling terms. Results were expanded to include references analysed in recent systematic and rapid reviews [[1](#_ENREF_1), [2](#_ENREF_2)]. The review was designed to support qualitative synthesis of principles for practical use in the consortium. We aimed to thoroughly cover concepts appearing in modelling guidance aimed at modellers. Author MRB did the search, study selection, and data extraction. We worked to limit risk of bias through pre-specification and simplicity. Studies in the form of reviews and guidelines were eligible for review and those discussing modelling conduct or reporting in the abstract or title were included. Studies were excluded if guidance to modellers was not presented in a list or table. The text of each list item or table row was eligible for analysis. Succinct statements were included for analysis, not elaborative text. Text was copy-pasted from PDF files to standardized study extraction spreadsheets.

## **Data analysis methods**

Authors coded the dataset individually (MRB, TCP, WAS, SJV) and jointly as a single entity (MGB, JIDH, MW), producing five independently coded sets of data. Coders practiced on a sample of 50 statements before working on the full dataset. Modelling guideline statements were coded with the following ordinal scale of importance scores: 1: not applicable, 2: not necessary, 3: important, 4: extremely important, and 5: obvious (i.e., merely restates principles regarded as universally agreed upon). Missing values were filled with score 1 (not applicable). We then ranked the scores to select the top few statements we collectively considered extremely important. First, statements were sorted by median score from high to low. Where multiple statements shared the same median value, we sorted secondarily on variance across the score sets, variance low to high. For the purpose of ranking by median, scores of 5 (obvious statements) were recoded with 0, so extremely important statements receiving scores of 4 were then ranked on top. This recode was only used in calculation of the median score for ranking purposes.

Our purpose was to develop a clear understanding of what guidance in existing literature would be useful to the Consortium to follow. To do this, we scored guidance statements by perceived importance to the Consortium. This allowed us to systematically identify relevant concepts as a basis for outlining our own principles. Importantly, the data (i.e. the statements extracted from the literature) were preserved entirely until they were distilled into the principles.

## **Results**

We identified 331 records with 288 unique studies relevant to modelling practices, of which 57 were included [[3-59](#_ENREF_3)] (Table 1). Search on four sources had given largely exclusive results, with each source contributing to included studies as the sole contributor in counts: 7 PubMed, 16 EQUATOR, 18 Dahabreh et al (2017) [[1](#_ENREF_1)], and 8 Egger et al (2017) [[2](#_ENREF_2)]. The remaining eight included studies were contributed in multiple sources. Of included studies, three were conducted by government health agencies [[9](#_ENREF_9), [14](#_ENREF_14), [1](#_ENREF_1)], one by a journal [[20](#_ENREF_20)], 17 by working groups [[7](#_ENREF_7), [10](#_ENREF_10), [15](#_ENREF_15), [22-24](#_ENREF_22), [26](#_ENREF_26), [28](#_ENREF_28), [31](#_ENREF_31), [34](#_ENREF_34), [35](#_ENREF_35), [41](#_ENREF_41), [44](#_ENREF_44), [48](#_ENREF_48), [52](#_ENREF_52), [55](#_ENREF_55), [59](#_ENREF_59)] (including professional societies and commissioned reports), and the remaining 36 by researchers (*Table*). Four studies [[20](#_ENREF_20), [24](#_ENREF_24), [9](#_ENREF_9), [17](#_ENREF_17)] made recognisable statements of commitment to their recommendations, such that either the authors or others were obligated to follow them. Altogether, studies contained 1054 succinct statements of modelling guidance that were included in the synthesis. Before coding, the dataset was segmented by roughly categorizing statements by what the each seemed to target for improvement. Statements were divided in categories: 23 Policy, 167 Problem, 172 Outcome, 194 Compare, 114 Sensitivity, 133 Data, 118 Methods, and 133 Other. Authors of guidance literature tended to focus on the modelling process from problem conceptualization up to outputs and the reporting of results, but less on the active role of policymakers in the modelling collaboration.

## **Table 1** Characteristics of included studies

| **Author (year) [reference]** | **Commitment to use** | **Organisation (if any)** | **Consensus method** | **People forming consensus** | **Non-author experts** | **Peer feedback** | **Systematic review** | **Grading tool** |
| --- | --- | --- | --- | --- | --- | --- | --- | --- |
| Abuelezam et al (2013)[[3](#_ENREF_3)] | No | — | Author discussion | 3 | 0 | No | Yes | No |
| Baldwin et al (2017)[[4](#_ENREF_4)] | No | — | Author discussion | 2 | 0 | No | No | No |
| Bennett et al (2012)[[5](#_ENREF_5)] | No | — | Author discussion | 2 | 0 | No | Yes | No |
| Bilcke et al (2011)[[6](#_ENREF_6)] | No | — | Author discussion | 4 | 0 | No | No | No |
| Briggs et al (2012)[[7](#_ENREF_7)] | No | ISPOR - SMDM Task Force-6 | Author discussion | 6^a^ | — | Yes | No | No |
| Burke et al (2014)[[8](#_ENREF_8)] | No | — | Author discussion | 4 | 0 | No | No | No |
| CADTH (2006)[[9](#_ENREF_9)] | Yes^b^ | Canadian Agency for Drugs and Technologies in Health (CADTH) | Author discussion | 7 | 6 | Yes | No | No |
| Caro et al (2014)[[10](#_ENREF_10)] | No | ISPOR - AMCP-NPC Good Practice Task Force | Author discussion | 7 | 48 | Yes | No | No |
| Carrasco et al (2013)[[11](#_ENREF_11)] | No | — | Author discussion | 6 | 0 | No | No | No |
| Chilcott et al (2010)[[12](#_ENREF_12)] | No | — | Author discussion | 8 | 12 | No | No | No |
| Chiou et al (2003)[[13](#_ENREF_13)] | No | — | Qualitative Analysis | 10 | 120 | Yes | Yes | Yes |
| Cleemput et al (2009)[[14](#_ENREF_14)] | No | Belgian Health Care Knowledge Centre | Author discussion | 4 | 12 | Yes | No | No |
| Clemens et al (1995)[[15](#_ENREF_15)] | No | Pharmaceutical Research and Manufacturers of America | Author discussion | 6 | — | Yes | No | No |
| Collins et al (2015)[[16](#_ENREF_16)] | No | — | Group discussion | 24 | 27 | Yes | Yes | No |
| Dahabreh et al (2016)[[17](#_ENREF_17)] | Yes^c^ | AHRQ and Tufts Evidence-based Practice Center | Author discussion | 4 | 28 | Yes | Yes | No |
| Detsky (1993)[[18](#_ENREF_18)] | No | — | Author discussion | 1 | 0 | Yes | No | No |
| Drummond et al (1993)[[19](#_ENREF_19)] | No | — | Author discussion | 4 | 0 | No | No | No |
| Drummond et al (1996)[[20](#_ENREF_20)] | Yes^d^ | BMJ Economic Evaluation Working Party | Group discussion | 17 | 15 | Yes | No | No |
| Dykstra et al (2015)[[21](#_ENREF_21)] | No | Model-Based Drug Development Consortium | Author discussion | 9 | 351 | Yes | No | No |
| Eddy et al (2012)[[22](#_ENREF_22)] | No | ISPOR - SMDM Task Force-7 | Author discussion | 6 ^a^ | — | Yes | No | No |
| Evers et al (2005)[[23](#_ENREF_23)] | No | Consensus on Health Economic Criteria | Delphi | 26 | 23 | No | No | No |
| Fry et al (2003)[[24](#_ENREF_24)] | Yes^b^ | Academy of Managed Care Pharmacy's Format Revision Committee | Author discussion | 3 | 6 | Yes | No | No |
| Goldhaber-Fiebert et al (2010)[[25](#_ENREF_25)] | No | — | Author discussion | 3 | 0 | No | No | No |
| Husereau et al (2013)[[26](#_ENREF_26)] | No | ISPOR Health Economic Evaluations Publication Guidelines Task Force | Delphi | 47 | 37 | Yes | Yes | No |
| Jackson (2010)[[27](#_ENREF_27)] | No | — | Author discussion | 1 | 0 | No | No | No |
| Janssens et al (2011)[[28](#_ENREF_28)] | No | Genetic Risk Prediction Studies Group | — | 25 | 20 | No | No | No |
| Kalil et al (2010)[[29](#_ENREF_29)] | No | — | Author discussion | 5 | 0 | No | Yes | No |
| Karnon et al (2007)[[30](#_ENREF_30)] | No | — | Author discussion | 7 | 0 | Yes | Yes | No |
| Karnon et al (2012)[[31](#_ENREF_31)] | No | ISPOR - SMDM Task Force-4 | Author discussion | 6 ^a^ | — | Yes | No | No |
| Kerr et al (2015)[[32](#_ENREF_32)] | No | — | Author discussion | 5 | 0 | No | No | No |
| Kostoulas et al (2017)[[33](#_ENREF_33)] | No | — | Author discussion | 8 | 0 | No | No | No |
| Liberati et al (1997)[[34](#_ENREF_34)] | No | EUR-ASSESS Subgroup on Methodology (commissioned) | Author discussion | 8 | 0 | No | No | No |
| LopezBastida et al (2010)[[35](#_ENREF_35)] | No | Evaluation and Planning Unit, Canary Islands Health Services (commissioned) | Author discussion | 7 | 0 | Yes | No | No |
| Luo et al (2016)[[36](#_ENREF_36)] | No | — | Delphi | 11 | — | No | No | No |
| McCabe et al (2000)[[37](#_ENREF_37)] | No | — | Author discussion | 2 | 0 | No | No | No |
| Nuijten et al (1998)[[38](#_ENREF_38)] | No | — | Author discussion | 8 | 0 | No | No | No |
| Olson et al (2003)[[39](#_ENREF_39)] | No | — | Author discussion | 4 | 20 | No | No | No |
| Philips et al (2006)[[40](#_ENREF_40)] | No | — | Author discussion | 5 | 8 | No | No | No |
| Pitman et al (2012)[[41](#_ENREF_41)] | No | ISPOR - SMDM Task Force-5 | Author discussion | 7 ^a^ | — | Yes | No | No |
| Poldrack et al (2008)[[42](#_ENREF_42)] | No | — | Author discussion | 6 | 0 | No | No | No |
| Ramos et al (2015)[[43](#_ENREF_43)] | No | — | Author discussion | 4 | 0 | No | Yes | No |
| Roberts et al (2012)[[44](#_ENREF_44)] | No | ISPOR - SMDM Task Force-2 | Author discussion | 6 ^a^ | — | Yes | No | No |
| Rodrigues et al (2004)[[45](#_ENREF_45)] | No | — | Author discussion | 5 | 0 | No | Yes | No |
| Scheiber (2017)[[46](#_ENREF_46)] | No | — | Author discussion | 1 | 0 | No | No | No |
| Sculpher et al (2004)[[47](#_ENREF_47)] | No | — | Author discussion | 8 | — | — | Yes | No |
| Siebert et al (2012)[[48](#_ENREF_48)] | No | ISPOR - SMDM Task Force-3 | Author discussion | 7 ^a^ | — | Yes | No | No |
| Soto (2002)[[49](#_ENREF_49)] | No | — | Author discussion | 1 | 0 | No | No | No |
| Spiegel et al (2004) [[50](#_ENREF_50)] ^e^ | No | — | — | — | — | — | — | Yes |
| Sterne et al (2009)[[51](#_ENREF_51)] | No | — | Author discussion | 8 | 0 | No | No | No |
| Stevens et al (2016)[[52](#_ENREF_52)] | No | GATHER working group | Author discussion | 19 | 118 | Yes | Yes | No |
| Stout et al (2009)[[53](#_ENREF_53)] | No | — | Author discussion | 5 | 0 | No | No | No |
| Subramanian et al (2010)[[54](#_ENREF_54)] | No | — | Author discussion | 2 | 0 | No | No | No |
| Ultsch et al (2016)[[55](#_ENREF_55)] | No | Robert Koch Institute (commissioned) | Vote | 20 | 0 | No | Yes | No |
| Ungar et al (2003)[[56](#_ENREF_56)] | No | — | Qualitative Analysis | — | 7 | No | No | Yes |
| van de Schoot et al (2017)[[57](#_ENREF_57)] | No | — | Delphi | 30 | 25 | No | No | No |
| Vegter et al (2008)[[58](#_ENREF_58)] | No | — | Author discussion | 6 | 0 | No | Yes | No |
| Weinstein et al (2003)[[59](#_ENREF_59)] | No | ISPOR Task Force on Good Research Practices | Author discussion | 7 | 0 | Yes | No | No |

AHRQ=US Agency for Healthcare Research and Quality. GATHER=Guidelines for Accurate and Transparent Health Estimates Reporting. ISPOR=International Society for Pharmacoeconomics and Outcomes Research.
^a^Authorship of guidelines rested with the working group, although consensus was broader across all working groups of the task force.
^b^Commitment was demonstrated in publishing updated guidelines from feedback after a period of practical use.
^c^Adopted by majority vote of the agency’s directors.
^d^Commitment was to a specified plan for pilot use of the guidelines.
^e^The QHES guidelines (Chiou 2003) were recaptured from their listing in this article on quality assessment. Duplications were not removed from the dataset.

## **Interrater reliability**

Interrater reliability was assessed by intraclass correlation coefficient (ICC) according to our purpose of identifying important statements, in our average judgement. ICC estimates were calculated in R (ver 3·6) using the package ‘psych’ (ver 1·8·12), based on a model of mean rating (k=5), consistency, and two-way mixed effects. The ICC of 0·69 (95%CI 0·66 - 0·72, n=1054) was in the moderate range. This indicates we should expect to have a mixture of more and less important statements in any bracket of ranked statements we might select in the synthesis process.

## **Word occurrence counts in guidance statements**

Word occurrence was counted in the dataset of 1054 guidance statements. Words having a count of 10 or more are shown in Table S below. Frequency is given per 1054 rows of the dataset. The guidance text dataset was processed with the natural language processing software spaCy (http://textanalysisonline.com/spacy-word-lemmatize) to obtain text will all words in their base forms. Words were counted in the lemmatised text using a tool for generating word clouds that also returns the numeric word count (<https://www.wordclouds.com>), excluding stop words.

## **Table S** Word occurrence counts in guidance dataset

| **Frequency** | **Count** | **Word** |
| --- | --- | --- |
| **Frequency** | **Count** | **Word** |
| **Frequency** | **Count** | **Word** |

| 0.482 | 508 | model |
| --- | --- | --- |
| 0.246 | 259 | data |
| 0.193 | 203 | use |
| 0.179 | 189 | analysis |
| 0.127 | 134 | study |
| 0.114 | 120 | describe |
| 0.107 | 113 | result |
| 0.096 | 101 | report |
| 0.095 | 100 | include |
| 0.095 | 100 | state |
| 0.088 | 93 | parameter |
| 0.084 | 89 | uncertainty |
| 0.081 | 85 | decision |
| 0.079 | 83 | method |
| 0.076 | 80 | justify |
| 0.073 | 77 | source |
| 0.067 | 71 | assumption |
| 0.067 | 71 | outcome |
| 0.065 | 69 | sensitivity |
| 0.064 | 67 | time |
| 0.057 | 60 | structure |
| 0.056 | 59 | estimate |
| 0.056 | 59 | value |
| 0.054 | 57 | alternative |
| 0.054 | 57 | variable |
| 0.054 | 57 | relevant |
| 0.053 | 56 | provide |
| 0.053 | 56 | cost |
| 0.050 | 53 | test |
| 0.047 | 50 | evidence |
| 0.046 | 49 | clinical |
| 0.045 | 47 | problem |
| 0.045 | 47 | e.g |
| 0.043 | 45 | validation |
| 0.043 | 45 | clearly |
| 0.042 | 44 | question |
| 0.041 | 43 | measure |
| 0.041 | 43 | disease |
| 0.041 | 43 | health |
| 0.041 | 43 | input |
| 0.040 | 42 | define |
| 0.040 | 42 | base |
| 0.039 | 41 | intervention |
| 0.039 | 41 | perspective |
| 0.039 | 41 | appropriate |
| 0.039 | 41 | give |
| 0.038 | 40 | treatment |
| 0.038 | 40 | available |
| 0.038 | 40 | choice |
| 0.037 | 39 | discuss |
| 0.037 | 39 | specify |
| 0.036 | 38 | possible |
| 0.035 | 37 | distribution |
| 0.035 | 37 | description |
| 0.035 | 37 | make |
| 0.034 | 36 | evaluation |
| 0.034 | 36 | population |
| 0.034 | 36 | effect |
| 0.033 | 35 | consider |
| 0.033 | 35 | number |
| 0.033 | 35 | risk |
| 0.033 | 35 | may |
| 0.032 | 34 | explain |
| 0.032 | 34 | assess |
| 0.031 | 33 | objective |
| 0.030 | 32 | horizon |
| 0.030 | 32 | output |
| 0.029 | 31 | important |
| 0.029 | 31 | research |
| 0.029 | 31 | identify |
| 0.029 | 31 | present |
| 0.028 | 30 | information |
| 0.028 | 30 | difference |
| 0.028 | 30 | detail |
| 0.028 | 30 | type |
| 0.028 | 29 | clear |
| 0.027 | 28 | statistical |
| 0.027 | 28 | prediction |
| 0.027 | 28 | modeling |
| 0.027 | 28 | compare |
| 0.026 | 27 | probability |
| 0.026 | 27 | incorporate |
| 0.026 | 27 | perform |
| 0.025 | 26 | individual |
| 0.025 | 26 | validity |
| 0.025 | 26 | process |
| 0.025 | 26 | can |
| 0.024 | 25 | limitation |
| 0.024 | 25 | choose |
| 0.024 | 25 | final |
| 0.024 | 25 | event |
| 0.023 | 24 | reflect |
| 0.023 | 24 | address |
| 0.023 | 24 | expert |
| 0.023 | 24 | key |
| 0.022 | 23 | characteristic |
| 0.022 | 23 | effectiveness |
| 0.022 | 23 | setting |
| 0.022 | 23 | range |
| 0.022 | 23 | need |
| 0.022 | 23 | term |
| 0.021 | 22 | whether |
| 0.021 | 22 | maker |
| 0.020 | 21 | consistent |
| 0.020 | 21 | miss |
| 0.020 | 21 | case |
| 0.019 | 20 | justification |
| 0.019 | 20 | structural |
| 0.019 | 20 | criterion |
| 0.019 | 20 | rationale |
| 0.019 | 20 | different |
| 0.019 | 20 | specific |
| 0.019 | 20 | interval |
| 0.019 | 20 | strategy |
| 0.019 | 20 | quality |
| 0.019 | 20 | option |
| 0.019 | 20 | impact |
| 0.018 | 19 | selection |
| 0.018 | 19 | document |
| 0.018 | 19 | evaluate |
| 0.018 | 19 | develop |
| 0.018 | 19 | target |
| 0.017 | 18 | approach |
| 0.017 | 18 | relate |
| 0.017 | 18 | random |
| 0.017 | 18 | well |
| 0.016 | 17 | heterogeneity |
| 0.016 | 17 | calibration |
| 0.016 | 17 | transition |
| 0.016 | 17 | comparison |
| 0.016 | 17 | reference |
| 0.016 | 17 | patient |
| 0.016 | 17 | within |
| 0.016 | 17 | cycle |
| 0.016 | 17 | scope |
| 0.016 | 17 | size |
| 0.016 | 17 | set |
| 0.016 | 17 | run |
| 0.015 | 16 | independent |
| 0.015 | 16 | simulation |
| 0.015 | 16 | economic |
| 0.015 | 16 | interest |
| 0.015 | 16 | subgroup |
| 0.015 | 16 | review |
| 0.015 | 16 | sample |
| 0.015 | 16 | derive |
| 0.015 | 16 | allow |
| 0.015 | 16 | rate |
| 0.015 | 16 | also |
| 0.015 | 16 | one |
| 0.014 | 15 | development |
| 0.014 | 15 | technique |
| 0.014 | 15 | practice |
| 0.014 | 15 | reason |
| 0.014 | 15 | care |
| 0.014 | 15 | bias |
| 0.014 | 15 | way |
| 0.013 | 14 | probabilistic |
| 0.013 | 14 | performance |
| 0.013 | 14 | participant |
| 0.013 | 14 | predictive |
| 0.013 | 14 | procedure |
| 0.013 | 14 | potential |
| 0.013 | 14 | resource |
| 0.013 | 14 | account |
| 0.013 | 14 | handle |
| 0.013 | 14 | and/or |
| 0.013 | 14 | author |
| 0.013 | 14 | metric |
| 0.013 | 14 | point |
| 0.013 | 14 | main |
| 0.012 | 13 | methodology |
| 0.012 | 13 | sufficient |
| 0.012 | 13 | predictor |
| 0.012 | 13 | multiple |
| 0.012 | 13 | software |
| 0.012 | 13 | validate |
| 0.012 | 13 | benefit |
| 0.012 | 13 | control |
| 0.012 | 13 | inform |
| 0.012 | 13 | change |
| 0.012 | 13 | trial |
| 0.012 | 13 | best |
| 0.012 | 13 | mean |
| 0.012 | 13 | i.e |
| 0.011 | 12 | consideration |
| 0.011 | 12 | interaction |
| 0.011 | 12 | transparent |
| 0.011 | 12 | assessment |
| 0.011 | 12 | conclusion |
| 0.011 | 12 | imputation |
| 0.011 | 12 | condition |
| 0.011 | 12 | statement |
| 0.011 | 12 | calibrate |
| 0.011 | 12 | complete |
| 0.011 | 12 | require |
| 0.011 | 12 | support |
| 0.011 | 12 | purpose |
| 0.011 | 12 | design |
| 0.011 | 12 | cohort |
| 0.011 | 12 | policy |
| 0.011 | 12 | regard |
| 0.011 | 12 | exist |
| 0.011 | 12 | must |
| 0.011 | 12 | take |
| 0.011 | 12 | new |
| 0.010 | 11 | confidence |
| 0.010 | 11 | explicitly |
| 0.010 | 11 | literature |
| 0.010 | 11 | particular |
| 0.010 | 11 | represent |
| 0.010 | 11 | discount |
| 0.010 | 11 | generate |
| 0.010 | 11 | previous |
| 0.010 | 11 | adequate |
| 0.010 | 11 | follow |
| 0.010 | 11 | select |
| 0.010 | 11 | enough |
| 0.010 | 11 | prior |
| 0.010 | 11 | group |
| 0.010 | 11 | face |
| 0.009 | 10 | appropriately |
| 0.009 | 10 | specification |
| 0.009 | 10 | implication |
| 0.009 | 10 | hypothesis |
| 0.009 | 10 | clinician |
| 0.009 | 10 | modelling |
| 0.009 | 10 | explicit |
| 0.009 | 10 | standard |
| 0.009 | 10 | external |
| 0.009 | 10 | internal |
| 0.009 | 10 | indicate |
| 0.009 | 10 | subject |
| 0.009 | 10 | primary |
| 0.009 | 10 | opinion |
| 0.009 | 10 | pathway |
| 0.009 | 10 | current |
| 0.009 | 10 | context |
| 0.009 | 10 | conduct |
| 0.009 | 10 | obtain |
| 0.009 | 10 | system |
| 0.009 | 10 | error |
| 0.009 | 10 | unit |
| 0.009 | 10 | show |
| 0.009 | 10 | list |

## **References to the supplementary appendix**

1. Dahabreh IJ, Chan JA, Earley A, Moorthy D, Avendano EE, Trikalinos TA et al. Modeling and simulation in the context of health technology assessment: Review of existing guidance, future research needs, and validity assessment: Agency for Healthcare Research and Quality, USA2017 Contract No.: Report No 16(17)-EHC020-EF.
2. Egger M, Johnson L, Althaus C, Schoni A, Salanti G, Low N et al. Developing WHO guidelines: time to formally include evidence from mathematical modelling studies. F1000Res. 2017;6:1584. doi:10.12688/f1000research.12367.2.
3. Abuelezam NN, Rough K, Seage GR, 3rd. Individual-based simulation models of HIV transmission: reporting quality and recommendations. PLoS One. 2013;8(9):e75624. doi:10.1371/journal.pone.0075624.
4. Baldwin SA, Larson MJ. An introduction to using Bayesian linear regression with clinical data. Behav Res Ther. 2017;98:58-75. doi:10.1016/j.brat.2016.12.016.
5. Bennett C, Manuel DG. Reporting guidelines for modelling studies. BMC Med Res Methodol. 2012;12:168. doi:10.1186/1471-2288-12-168.
6. Bilcke J, Beutels P, Brisson M, Jit M. Accounting for methodological, structural, and parameter uncertainty in decision-analytic models: a practical guide. Med Decis Making. 2011;31(4):675-92. doi:10.1177/0272989X11409240.
7. Briggs AH, Weinstein MC, Fenwick EA, Karnon J, Sculpher MJ, Paltiel AD. Model parameter estimation and uncertainty: a report of the ISPOR-SMDM Modeling Good Research Practices Task Force--6. Value Health. 2012;15(6):835-42. doi:10.1016/j.jval.2012.04.014.
8. Burke DL, Billingham LJ, Girling AJ, Riley RD. Meta-analysis of randomized phase II trials to inform subsequent phase III decisions. Trials. 2014;15:346. doi:10.1186/1745-6215-15-346.
9. CADTH. Guidelines for the economic evaluation of health technologies: Canada. Canadian Agency for Drugs and Technologies in Health. 2006(3rd Edition).
10. Caro JJ, Eddy DM, Kan H, Kaltz C, Patel B, Eldessouki R et al. Questionnaire to assess relevance and credibility of modeling studies for informing health care decision making: an ISPOR-AMCP-NPC Good Practice Task Force report. Value Health. 2014;17(2):174-82. doi:10.1016/j.jval.2014.01.003.
11. Carrasco LR, Jit M, Chen MI, Lee VJ, Milne GJ, Cook AR. Trends in parameterization, economics and host behaviour in influenza pandemic modelling: a review and reporting protocol. Emerg Themes Epidemiol. 2013;10(1):3. doi:10.1186/1742-7622-10-3.
12. Chilcott J, Tappenden P, Rawdin A, Johnson M, Kaltenthaler E, Paisley S et al. Avoiding and identifying errors in health technology assessment models: qualitative study and methodological review. Health Technol Assess. 2010;14(25):iii-iv, ix-xii, 1-107. doi:10.3310/hta14250.
13. Chiou CF, Hay JW, Wallace JF, Bloom BS, Neumann PJ, Sullivan SD et al. Development and validation of a grading system for the quality of cost-effectiveness studies. Med Care. 2003;41(1):32-44. doi:10.1097/01.MLR.0000039824.73620.E5.
14. Cleemput I, van Wilder P, Huybrechts M, Vrijens F. Belgian methodological guidelines for pharmacoeconomic evaluations: toward standardization of drug reimbursement requests. Value Health. 2009;12(4):441-9. doi:10.1111/j.1524-4733.2008.00469.x.
15. Clemens K, Townsend R, Luscombe F, Mauskopf J, Osterhaus J, Bobula J. Methodological and conduct principles for pharmacoeconomic research. Pharmaceutical Research and Manufacturers of America. Pharmacoeconomics. 1995;8(2):169-74.
16. Collins GS, Reitsma JB, Altman DG, Moons KG. Transparent reporting of a multivariable prediction model for individual prognosis or diagnosis (TRIPOD): the TRIPOD statement. BMJ. 2015;350:g7594. doi:10.1136/bmj.g7594.
17. Dahabreh IJ, Trikalinos TA, Balk EM, Wong JB. Recommendations for the conduct and reporting of modeling and simulation studies in health technology assessment. Ann Intern Med. 2016;165(8):575-81. doi:10.7326/m16-0161.
18. Detsky AS. Guidelines for economic analysis of pharmaceutical products: a draft document for Ontario and Canada. Pharmacoeconomics. 1993;3(5):354-61.
19. Drummond M, Brandt A, Luce B, Rovira J. Standardizing methodologies for economic evaluation in health care. Practice, problems, and potential. Int J Technol Assess Health Care. 1993;9(1):26-36.
20. Drummond MF, Jefferson TO. Guidelines for authors and peer reviewers of economic submissions to the BMJ. The BMJ Economic Evaluation Working Party. BMJ. 1996;313(7052):275-83.
21. Dykstra K, Mehrotra N, Tornoe CW, Kastrissios H, Patel B, Al-Huniti N et al. Reporting guidelines for population pharmacokinetic analyses. J Clin Pharmacol. 2015;55(8):875-87. doi:10.1002/jcph.532.
22. Eddy DM, Hollingworth W, Caro JJ, Tsevat J, McDonald KM, Wong JB. Model transparency and validation: a report of the ISPOR-SMDM Modeling Good Research Practices Task Force--7. Value Health. 2012;15(6):843-50. doi:10.1016/j.jval.2012.04.012.
23. Evers S, Goossens M, de Vet H, van Tulder M, Ament A. Criteria list for assessment of methodological quality of economic evaluations: Consensus on Health Economic Criteria. Int J Technol Assess Health Care. 2005;21(2):240-5.
24. Fry RN, Avey SG, Sullivan SD. The Academy of Managed Care Pharmacy format for formulary submissions: an evolving standard--a Foundation for Managed Care Pharmacy Task Force report. Value Health. 2003;6(5):505-21. doi:10.1046/j.1524-4733.2003.65327.x.
25. Goldhaber-Fiebert JD, Stout NK, Goldie SJ. Empirically evaluating decision-analytic models. Value Health. 2010;13(5):667-74. doi:10.1111/j.1524-4733.2010.00698.x.
26. Husereau D, Drummond M, Petrou S, Carswell C, Moher D, Greenberg D et al. Consolidated health economic evaluation reporting standards (CHEERS) statement. BMJ. 2013;346:f1049. doi:10.1136/bmj.f1049.
27. Jackson DL. Reporting results of latent growth modeling and multilevel modeling analyses: Some recommendations for rehabilitation psychology. Rehabilitation Psychology. 2010;55(3):272-85. doi:10.1037/a0020462.
28. Janssens AC, Ioannidis JP, van Duijn CM, Little J, Khoury MJ. Strengthening the reporting of genetic risk prediction studies: the GRIPS statement. PLoS Med. 2011;8(3):e1000420. doi:10.1371/journal.pmed.1000420.
29. Kalil AC, Mattei J, Florescu DF, Sun J, Kalil RS. Recommendations for the assessment and reporting of multivariable logistic regression in transplantation literature. Am J Transplant. 2010;10(7):1686-94. doi:10.1111/j.1600-6143.2010.03141.x.
30. Karnon J, Goyder E, Tappenden P, McPhie S, Towers I, Brazier J et al. A review and critique of modelling in prioritising and designing screening programmes. Health Technol Assess. 2007;11(52):iii-iv, ix-xi, 1-145.
31. Karnon J, Stahl J, Brennan A, Caro JJ, Mar J, Moller J. Modeling using discrete event simulation: a report of the ISPOR-SMDM Modeling Good Research Practices Task Force--4. Value Health. 2012;15(6):821-7. doi:10.1016/j.jval.2012.04.013.
32. Kerr KF, Meisner A, Thiessen-Philbrook H, Coca SG, Parikh CR. RiGoR: reporting guidelines to address common sources of bias in risk model development. Biomark Res. 2015;3(1):2. doi:10.1186/s40364-014-0027-7.
33. Kostoulas P, Nielsen SS, Branscum AJ, Johnson WO, Dendukuri N, Dhand NK et al. STARD-BLCM: standards for the reporting of diagnostic accuracy studies that use Bayesian latent class models. Prev Vet Med. 2017;138:37-47. doi:https://doi.org/10.1016/j.prevetmed.2017.01.006.
34. Liberati A, Sheldon TA, Banta HD. EUR-ASSESS project subgroup report on methodology: methodological guidance for the conduct of health technology assessment. Int J Technol Assess Health Care. 1997;13(2):186-219.
35. Lopez-Bastida J, Oliva J, Antonanzas F, Garcia-Altes A, Gisbert R, Mar J et al. Spanish recommendations on economic evaluation of health technologies. Eur J Health Econ. 2010;11(5):513-20. doi:10.1007/s10198-010-0244-4.
36. Luo W, Phung D, Tran T, Gupta S, Rana S, Karmakar C et al. Guidelines for developing and reporting machine learning predictive models in biomedical research: a multidisciplinary view. J Med Internet Res. 2016;18(12):e323. doi:10.2196/jmir.5870.
37. McCabe C, Dixon S. Testing the validity of cost-effectiveness models. Pharmacoeconomics. 2000;17(5):501-13.
38. Nuijten MJC, Pronk MH, Brorens MJA, Hekster YA, Lockefeer JHM, de Smet PAGM et al. Reporting format for economic evaluation part II: focus on modelling studies. Pharmacoeconomics. 1998;14(3):259-68. doi:10.2165/00019053-199814030-00003.
39. Olson BM, Armstrong EP, Grizzle AJ, Nichter MA. Industry's perception of presenting pharmacoeconomic models to managed care organizations. J Manag Care Pharm. 2003;9(2):159-67. doi:10.18553/jmcp.2003.9.2.159.
40. Philips Z, Bojke L, Sculpher M, Claxton K, Golder S. Good practice guidelines for decision-analytic modelling in health technology assessment: a review and consolidation of quality assessment. Pharmacoeconomics. 2006;24(4):355-71.
41. Pitman R, Fisman D, Zaric GS, Postma M, Kretzschmar M, Edmunds J et al. Dynamic transmission modeling: a report of the ISPOR-SMDM Modeling Good Research Practices Task Force--5. Value Health. 2012;15(6):828-34. doi:10.1016/j.jval.2012.06.011.
42. Poldrack RA, Fletcher PC, Henson RN, Worsley KJ, Brett M, Nichols TE. Guidelines for reporting an fMRI study. Neuroimage. 2008;40(2):409-14. doi:10.1016/j.neuroimage.2007.11.048.
43. Ramos MC, Barton P, Jowett S, Sutton AJ. A systematic review of research guidelines in decision-analytic modeling. Value Health. 2015;18(4):512-29. doi:10.1016/j.jval.2014.12.014.
44. Roberts M, Russell LB, Paltiel AD, Chambers M, McEwan P, Krahn M. Conceptualizing a model: a report of the ISPOR-SMDM Modeling Good Research Practices Task Force-2. Med Decis Making. 2012;32(5):678-89. doi:10.1177/0272989X12454941.
45. Rodrigues G, Lock M, D'Souza D, Yu E, Van Dyk J. Prediction of radiation pneumonitis by dose - volume histogram parameters in lung cancer--a systematic review. Radiother Oncol. 2004;71(2):127-38. doi:10.1016/j.radonc.2004.02.015.
46. Schreiber JB. Latent class analysis: an example for reporting results. Research in Social and Administrative Pharmacy. 2017;13(6):1196-201. doi:https://doi.org/10.1016/j.sapharm.2016.11.011.
47. Sculpher MJ, Pang FS, Manca A, Drummond MF, Golder S, Urdahl H et al. Generalisability in economic evaluation studies in healthcare: a review and case studies. Health Technol Assess. 2004;8(49):iii-iv, 1-192.
48. Siebert U, Alagoz O, Bayoumi AM, Jahn B, Owens DK, Cohen DJ et al. State-transition modeling: a report of the ISPOR-SMDM Modeling Good Research Practices Task Force--3. Value Health. 2012;15(6):812-20. doi:10.1016/j.jval.2012.06.014.
49. Soto J. Health economic evaluations using decision analytic modeling: principles and practices—utilization of a checklist to their development and appraisal. Int J Technol Assess Health Care. 2002;18(1):94-111. doi:undefined.
50. Spiegel BM, Targownik LE, Kanwal F, Derosa V, Dulai GS, Gralnek IM et al. The quality of published health economic analyses in digestive diseases: a systematic review and quantitative appraisal. Gastroenterology. 2004;127(2):403-11.
51. Sterne JA, White IR, Carlin JB, Spratt M, Royston P, Kenward MG et al. Multiple imputation for missing data in epidemiological and clinical research: potential and pitfalls. BMJ. 2009;338:b2393. doi:10.1136/bmj.b2393.
52. Stevens GA, Alkema L, Black RE, Boerma JT, Collins GS, Ezzati M et al. Guidelines for Accurate and Transparent Health Estimates Reporting: the GATHER statement. The Lancet. 2016;388(10062):e19-e23. doi:10.1016/s0140-6736(16)30388-9.
53. Stout NK, Knudsen AB, Kong CY, McMahon PM, Gazelle GS. Calibration methods used in cancer simulation models and suggested reporting guidelines. Pharmacoeconomics. 2009;27(7):533-45. doi:10.2165/11314830-000000000-00000.
54. Subramanian J, Simon R. Gene expression-based prognostic signatures in lung cancer: ready for clinical use? J Natl Cancer Inst. 2010;102(7):464-74. doi:10.1093/jnci/djq025.
55. Ultsch B, Damm O, Beutels P, Bilcke J, Bruggenjurgen B, Gerber-Grote A et al. Methods for health economic evaluation of vaccines and immunization decision frameworks: a consensus framework from a European vaccine economics community. Pharmacoeconomics. 2016;34(3):227-44. doi:10.1007/s40273-015-0335-2.
56. Ungar WJ, Santos MT. The Pediatric Quality Appraisal Questionnaire: an instrument for evaluation of the pediatric health economics literature. Value Health. 2003;6(5):584-94. doi:10.1046/j.1524-4733.2003.65253.x.
57. van de Schoot R, Sijbrandij M, Winter SD, Depaoli S, Vermunt JK. The GRoLTS-Checklist: guidelines for reporting on latent trajectory studies. Structural Equation Modeling: A Multidisciplinary Journal. 2016;24(3):451-67. doi:10.1080/10705511.2016.1247646.
58. Vegter S, Boersma C, Rozenbaum M, Wilffert B, Navis G, Postma MJ. Pharmacoeconomic evaluations of pharmacogenetic and genomic screening programmes: a systematic review on content and adherence to guidelines. Pharmacoeconomics. 2008;26(7):569-87.
59. Weinstein MC, O'Brien B, Hornberger J, Jackson J, Johannesson M, McCabe C et al. Principles of good practice for decision analytic modeling in health-care evaluation: report of the ISPOR Task Force on Good Research Practices--Modeling Studies. Value Health. 2003;6(1):9-17.
